# Comparative Nutritional Profiles of Three Edible Algae Collected From Peruvian Marine and Freshwater Environments

**DOI:** 10.1155/ijfo/2170707

**Published:** 2026-11-14

**Authors:** Liliana Asunción Sumarriva-Bustinza, Zaida Pumacayo-Sánchez, David Choque-Quispe, Nadia Chávez-Sumarriva, Ligia Isaida Rosaura Gutierrez-Deza, José Carlos Ayuque-Rojas, Janeth Bertha Mariño-Arroyo, Lucila Amelia De-La-Cruz-Rojas, Russbelt Yaulilahua-Huacho

**Affiliations:** ^1^ Faculty of Sciences National University of Education Enrique Guzman and Valle Lima Peru; ^2^ Department of Agroindustrial Engineering National University of Jose Maria Arguedas Andahuaylas Peru; ^3^ Faculty of Environmental Sciences Scientific University of South Lima Peru; ^4^ Faculty of Engineering Sciences National University of Jose Maria Arguedas Andahuaylas Peru; ^5^ Autonomous National University of Tayacaja Daniel Hernández Morillo Huancavelica Peru; ^6^ Faculty of Engineering Sciences National University of Huancavelica Huancavelica Peru

**Keywords:** amino acid quality, edible algae, food safety, mineral accumulation, nutritional composition, Peru

## Abstract

Edible algae are potentially nutrient‐dense food materials, but their composition varies with taxonomy, habitat, season, and processing. This study compared the proximate composition, gross energy, detergent‐fiber fractions, selected vitamins, carotenoids, minerals, and amino acid profiles of *Gigartina chamissoi*, *Monostroma cuaternaria*, and *Cladophora glomerata* collected from three Peruvian water bodies. Three independent biological samples were analyzed per taxon. *M. cuaternaria* had the highest crude protein concentration (31.97 ± 0.41 g/100 g dry weight [DW]) and measured adult indispensable amino acid total (230.4 mg/g protein). Its amino acid profile produced the highest descriptive essential amino acid index (0.994), although leucine was the first limiting amino acid in all three biomasses relative to the FAO adult reference pattern. *G. chamissoi* had the highest nitrogen‐free extract (63.60 ± 1.55 g/100 g DW) and niacin (4.040 ± 0.180 mg/100 g DW). *C. glomerata* contained the highest ash (32.09 ± 0.52 g/100 g DW), acid detergent fiber (27.90 ± 0.72 g/100 g DW), cellulose (23.74 ± 0.75 g/100 g DW), calcium (118.80 ± 2.74 mg/g DW), magnesium (109.00 ± 2.36 mg/g DW), and zinc (4738 ± 96 *μ*g/g DW). At the measured concentration, approximately 5.28 g of dried *C. glomerata* would contain 25 mg total zinc, indicating that its high mineral concentration should not be interpreted solely as a nutritional advantage. Because each taxon was obtained from a different location during one sampling period, the results characterize the sampled biomasses rather than universal species‐level profiles. Mineral bioaccessibility, contaminant screening, repeated seasonal sampling, and processing studies are required before food or fortification applications can be recommended.

## 1. Introduction

Edible macroalgae have long contributed to the food systems of coastal and highland communities in Peru. Archeological and ethnobotanical evidence indicates that algae were collected and consumed by pre‐Columbian populations as accessible sources of food and other useful materials [1]. This traditional use continues in several regions, where marine and freshwater algae are harvested for household consumption and local trade. Among the taxa used in Peru are *Gigartina chamissoi*, currently recognized in much of the taxonomic literature as *Chondracanthus chamissoi*, *Monostroma cuaternaria*, and *Cladophora glomerata*. These algae occur in contrasting marine, lacustrine, and riverine environments and may therefore display substantial differences in their biochemical composition.

Macroalgae are increasingly investigated as sustainable food materials because they can provide protein, amino acids, dietary fiber, minerals, vitamins, pigments, and structurally diverse polysaccharides while requiring little or no agricultural land [2, 3]. Their nutritional composition, however, varies considerably among taxonomic groups and is influenced by habitat, season, water chemistry, maturity, processing, and analytical method. Red algae frequently contain sulfated galactans, including carrageenan‐type polysaccharides, whereas green algae contain structurally distinct cell wall polysaccharides such as ulvan and cellulose [4, 5]. Freshwater filamentous algae such as *Cladophora* may also accumulate substantial quantities of minerals through binding sites associated with hydroxyl, carboxyl, and other functional groups in their cell walls [6, 7]. Consequently, nutritional characteristics reported for one algal species or habitat cannot be generalized to all edible algae.

Previous investigations have demonstrated the broad nutritional variability of edible macroalgae. Ortiz et al. [8] reported that *Ulva lactuca* and *Durvillaea antarctica* contained substantial dietary fiber fractions and nutritionally relevant amino acids, although their composition differed between species and morphological structures. Similarly, Ortiz et al. [9] found marked differences in protein, lipid, carbohydrate, amino acid, and pigment composition among *Codium fragile*, *Gracilaria chilensis*, and *Macrocystis pyrifera*. Yaich et al. [10] reported that *U. lactuca* contained approximately 54% fiber, 19.6% minerals, and 8.5% protein, illustrating the fiber‐ and mineral‐rich composition commonly associated with green macroalgae. These studies show that comparison with related algae is necessary to determine whether measured values are higher than, lower than, or consistent with previously reported nutritional profiles.

Research on Peruvian seaweeds has also expanded in recent years. Avila‐Peltroche and Padilla‐Vallejos [11] documented the diversity, utilization, and economic relevance of seaweed resources along the Peruvian coast. Arakaki et al. [12] examined the morphological and phylogenetic diversity of *Chondracanthus chamissoi*, an edible red alga of considerable cultural and commercial importance. More recently, Arakaki and Ramos [13] demonstrated substantial variation in the proximate composition, amino acid composition, and fatty acid profiles of edible seaweeds collected from the Peruvian coast. Their findings indicated that nutritional characteristics differed among taxonomic groups and supported the need for species‐ and sample‐specific characterization. Nevertheless, fewer comparative data are available for traditionally consumed algae collected from contrasting Peruvian marine and freshwater environments, particularly when mineral composition, detergent‐fiber fractions, selected vitamins, carotenoids, and comprehensive amino acid profiles are considered together.

The three algae examined in the present study represent distinct ecological and structural groups. *G. chamissoi* is a marine red alga associated with a polysaccharide‐rich thallus and is used as food and as a source of hydrocolloids. Red algae related to this taxon generally have low lipid concentrations but may contain appreciable protein, minerals, and carbohydrates [14, 15]. *M. cuaternaria* is a sheet‐forming green alga for which comparatively limited compositional information is available, although related green algae frequently contain substantial protein, essential amino acids, pigments, and dietary fiber [16]. *C. glomerata* is a freshwater filamentous green alga with a cellulose‐rich cell wall structure and considerable capacity to accumulate minerals from the surrounding environment [7, 17]. These differences provide a relevant basis for comparing the nutritional characteristics of the collected biomasses but do not, by themselves, establish causal effects of taxonomy or habitat.

Mineral profiling is especially important when algae are evaluated as potential food materials. Macroalgae can contain nutritionally relevant concentrations of Ca, Mg, K, Fe, Zn, Cu, Mn, and Se, but mineral accumulation is influenced strongly by local environmental conditions [2, 3]. The same binding mechanisms that promote the accumulation of essential minerals may also favor the uptake of potentially toxic elements. Therefore, a high total mineral concentration should not automatically be interpreted as evidence of nutritional superiority or food safety. Mineral results must be considered in relation to dietary intake, bioaccessibility, sampling location, analytical quality control, and the potential coaccumulation of undesirable elements. This consideration is particularly relevant for freshwater *Cladophora*, which is recognized for its capacity to bind and concentrate dissolved metals [6, 7].

Protein concentration alone is similarly insufficient for evaluating algal protein quality. The nutritional value of a protein depends on the concentrations and balance of indispensable amino acids relative to human requirements. Earlier studies of edible algae have demonstrated considerable variation in lysine, leucine, sulfur‐containing amino acids, and other indispensable amino acids [8, 16]. A comprehensive amino acid profile, together with total essential amino acids, the essential amino acid index (EAAI), and the chemical score, provides a more informative assessment than the measurement of a single amino acid. Accordingly, the present study evaluated a broad set of amino acids rather than restricting the analysis to leucine or lysine.

Although antioxidant activity and nonnutrient bioactive compounds are important aspects of algal functionality, their determination requires extraction‐ and assay‐specific protocols that were outside the analytical scope of the present investigation. The study instead focused on directly measured nutritional variables: proximate composition, gross energy, Van Soest fiber fractions, macro‐ and trace minerals, selected B vitamins, carotenoids, and a comprehensive amino acid profile. Antioxidant activity, phenolic compounds, mineral bioaccessibility, and contaminant screening are identified as priorities for subsequent research rather than being estimated from literature values.

Accordingly, the objective of this study was to compare the nutritional composition of *G. chamissoi*, *M. cuaternaria*, and *C. glomerata* collected from three Peruvian water bodies by determining their proximate composition, gross energy, fiber fractions, mineral concentrations, selected vitamins, carotenoids, amino acid profiles, and protein‐quality indices. It was hypothesized that the three sampled algal biomasses would exhibit different compositional patterns corresponding to their contrasting structural and environmental characteristics. Because each taxon was obtained from a different location during one sampling period, the results are interpreted as a comparative description of the collected biomasses rather than as definitive species‐wide nutritional profiles. The resulting dataset provides a foundation for future multisite, multiseason studies incorporating mineral bioaccessibility, contaminant screening, antioxidant activity, sensory acceptability, and food‐processing performance.

## 2. Materials and Methods

### 2.1. Study Design

A comparative laboratory study was conducted to characterize the nutritional composition of three edible algal biomasses collected from contrasting aquatic environments in Peru: *Chondracanthus chamissoi* (syn. *G. chamissoi*), *Monostroma quaternarium*, and *C. glomerata*. The analyses included proximate composition, gross energy, detergent‐fiber fractions, macro‐ and trace minerals, selected B vitamins, total carotenoids, amino acid composition, and protein‐quality indices. Each taxon was represented by three independently collected biological samples, which were transported, processed, and analyzed separately.

Because each taxon was obtained from a different water body, taxon and collection location were not independently crossed in the sampling design. Therefore, the study was designed to compare the collected algal biomasses, and observed differences were not attributed exclusively to species identity.

### 2.2. Study Areas and Sample Collection

The algal samples were collected in Peru between May and August 2024. *G. chamissoi* was collected from coastal waters near Ancón, Lima; *M. cuaternaria* was collected from Lake Titicaca, Puno; and *C. glomerata* was collected from the Urubamba River, Cusco. Available collection area, sampling period, replication, transport, and voucher status information are provided in Table S1. Exact collection dates, geographic coordinates, elevations or collection depths, and contemporaneous water‐quality measurements were unavailable. Representative images illustrating the macroscopic appearance of the three algal taxa investigated in this study are provided in Figure S1.

For each taxon, three spatially independent biological samples were collected from separate thalli, or algal patches located at least 10 m apart. Approximately 300 g of fresh biomass was collected for each biological replicate to provide sufficient material for sample preparation and the planned compositional analyses. Samples obtained from different collection points were kept separate and were not pooled. Marine biomass was collected manually during low tide or shallow submersion, whereas freshwater biomass was collected manually from accessible submerged substrates using clean equipment. Equipment used to collect samples intended for mineral analysis was acid‐washed before use.

Immediately after collection, visible sediment, associated organisms, and epiphytic material were removed by rinsing the biomass with water from the collection site. Each sample was placed separately in a clean, labeled polyethylene container and transported to the laboratory in an insulated container at approximately 4^°^C ± 2^°^C. The interval between collection and initial processing did not exceed 12 h.

### 2.3. Collection Authorization, Biodiversity Compliance, and Voucher Deposition

No specific collection permit or prior authorization from the Servicio Nacional Forestal y de Fauna Silvestre (SERFOR) or another competent regional or maritime authority was required because the algal material was collected from nonprotected areas for noncommercial scientific research. None of the sampled taxa was legally protected, and no material was obtained from a protected population or restricted access area. Collection and scientific use of the biological material were conducted in accordance with the applicable institutional and Peruvian biodiversity requirements.

Taxonomic identification was performed using morphological characteristics and standard taxonomic keys. Fresh specimens were photographed, and relevant field information, including the collection location, collection date, habitat, and morphological characteristics, was documented. However, representative specimens were not deposited in a formally recognized herbarium or biological collection, and voucher or accession numbers are therefore unavailable.

### 2.4. Sample Preparation

Each biological replicate was processed separately. Following taxonomic identification, the algal biomass was rinsed thoroughly with distilled water to remove residual salts, sediment, epiphytes, and other surface debris. Excess surface water was removed using clean absorbent paper. The samples were predried at room temperature (approximately 25^°^C ± 2^°^C) for 24 h in a clean, shaded, and well‐ventilated environment. They were subsequently oven‐dried in a Memmert universal oven at 60°C for approximately 48 h or until constant mass was achieved. Constant mass was defined as a difference of less than 0.01 g between two consecutive measurements taken at 2‐h intervals.

The dried biomass was ground using a Wiley Mill (Thomas Model 3, Thomas Scientific, United States) and passed through a 40‐mesh sieve (approximately 425 *μ*m). The powdered samples were transferred to airtight, light‐protected containers and stored at 4°C until analysis. All compositional results were expressed on a dry weight (DW) basis unless otherwise specified. The initial moisture content of the fresh biomass was approximately 82%; however, moisture was measured separately for each biological replicate, and the replicate‐specific value was used for DW conversion.

### 2.5. Proximate Composition and Gross Energy

Moisture, crude protein, crude fat, ash, and crude fiber were determined according to AOAC International procedures [18]. All measurements were performed separately for each biological replicate. Residual moisture was determined by drying approximately 2.0 g of the ground sample at 105^°^C ± 2^°^C until constant mass. Constant mass was defined as a difference of less than 0.01 g between two consecutive measurements obtained after an additional 1 h of drying.

Crude protein was determined using the Kjeldahl method. Approximately 0.5 g of dried sample was digested with concentrated sulfuric acid in the presence of a Kjeldahl catalyst mixture. After digestion, the sample was alkalinized, distilled, and titrated. Total nitrogen was multiplied by a conversion factor of 6.25, and the result was expressed as grams per 100 g DW:

Crude protein g100g DW=total nitrogen g100g DW×6.25.

Because validated species‐specific nitrogen‐to‐protein conversion factors were unavailable, the resulting values were reported as conventional crude protein estimates. These estimates may include a contribution from nonprotein nitrogen naturally present in algal biomass.

Crude fat was determined by Soxhlet extraction. Approximately 2.0 g of dried sample was placed in a cellulose extraction thimble and extracted with petroleum ether, with a boiling range of 40°C–60°C, for 6 h. After extraction, the solvent was removed, and the flask containing the lipid residue was dried at 105°C, cooled in a desiccator, and weighed. Crude fat was expressed as grams per 100 g DW.

Ash was determined by placing approximately 2.0 g of dried sample in a preweighed porcelain crucible and incinerating it in a muffle furnace at 550^°^C ± 10^°^C for approximately 6 h, or until a light‐gray residue of constant mass was obtained. The crucible was cooled in a desiccator before weighing, and ash content was expressed as grams per 100 g DW.

Crude fiber was measured by sequential acid and alkaline digestion. Approximately 1.0 g of defatted sample was boiled for 30 min in 1.25% sulfuric acid, filtered, washed, and subsequently boiled for 30 min in 1.25% sodium hydroxide. The recovered residue was washed, dried at 105°C to constant mass, weighed, incinerated at 550°C, and reweighed. Crude fiber was calculated from the difference between the dried residue and the ash remaining after incineration and was expressed as grams per 100 g DW.

Nitrogen‐free extract (Nifex) was calculated by difference:

Nifex g100g DW=100−crude protein+crude fat+crude fiber+ash.

Nifex was interpreted strictly as a calculated residual fraction and not as a direct measurement of soluble carbohydrates, total carbohydrates, individual polysaccharides, or carrageenan.

Gross energy was determined using an IKA C2000 oxygen bomb calorimeter (IKA‐Werke GmbH & Co. KG, Staufen, Germany). Approximately 0.500–1.000 g of dried and homogenized sample were accurately weighed, compressed into a pellet, placed in the combustion crucible, and combusted under an oxygen atmosphere according to the manufacturer′s operating procedure. The calorimeter was calibrated before sample analysis using certified benzoic acid with a certified heat of combustion of approximately 26.45 kJ/g. Corrections for the ignition wire and acid formation were applied when required by the operating method. Gross energy was initially obtained in kilojoules per gram DW and converted to kilocalories per gram DW using

Gross energykcalgDW=gross energy kJgDW÷4.184.

### 2.6. Detergent‐Fiber Fractionation

Neutral detergent fiber (NDF), acid detergent fiber (ADF), and acid detergent lignin (ADL) were determined using the sequential detergent procedure of Goering and Van Soest [19], with correction for residual ash because of the high mineral content of algal biomass. Approximately 0.50 g of dried and ground sample was weighed into an ANKOM F57 filter bag and analyzed using an ANKOM 200 Fiber Analyzer (ANKOM Technology, Macedon, New York, United States).

NDF was determined following digestion in neutral detergent solution. The recovered residue consisted predominantly of hemicellulose, cellulose, lignin, and detergent‐insoluble mineral matter. ADF was subsequently determined by digestion in acid detergent solution and represented predominantly cellulose, lignin, and acid‐insoluble mineral matter. ADL was determined by treating the ADF residue with 72% sulfuric acid. Following each applicable determination, the remaining residue was incinerated at 550°C to quantify residual ash. NDF, ADF, and ADL were therefore reported on an ash‐free basis as NDFom, ADFom, and ADL, respectively, where “om” indicates that residual inorganic matter was excluded. Blank filter bag corrections were applied to all gravimetric measurements.

Hemicellulose and cellulose concentrations were calculated separately for each biological replicate before calculation of the species‐level mean and standard deviation:

Hemicellulose g100g DW=NDFom−ADFom,

Cellulose g100g DW=ADFom−ADL.

The following fiber composition ratios were also calculated independently for each biological replicate:

NDF:ADF ratio=NDFom÷ADFom,

Hemicellulose:NDF ratio=hemicellulose÷NDFom,

Cellulose:ADF ratio=cellulose÷ADFom,

Lignin:ADF ratio=ADL÷ADFom.

Total structural carbohydrate was calculated as the sum of the hemicellulose and cellulose fractions:

Structural carbohydrate g100g DW=hemicellulose+cellulose.

All fiber fractions were expressed as grams per 100 g DW, whereas the derived ratios were dimensionless. Calculations were performed at the individual biological replicate level rather than by subtracting or dividing the species‐level means.

### 2.7. Mineral Analysis

#### 2.7.1. Sample Digestion

Mineral analysis was performed separately for each biological replicate. Approximately 0.500 g of dried and homogenized algal biomass was accurately weighed into an acid‐cleaned digestion vessel. Each sample was mixed with 12 mL of concentrated nitric acid (65%–70%, trace‐metal grade) and 4 mL of concentrated perchloric acid (70%, analytical grade), corresponding to a nitric acid‐to‐perchloric acid ratio of 3:1 (v/v). The samples were allowed to predigest at room temperature for approximately 30 min to reduce excessive foaming during heating.

Digestion was conducted on a temperature‐controlled digestion block inside a dedicated perchloric acid fume hood. The samples were initially heated at approximately 120°C for 30 min and then at 180°C for approximately 90–120 min, or until the evolution of dense fumes had ceased and a clear or nearly clear digest was obtained. The digests were not allowed to evaporate completely to dryness. After cooling to room temperature, each digest was quantitatively transferred to an acid‐cleaned 50‐mL volumetric flask and diluted to volume with ultrapure water. When necessary, the diluted digests were passed through acid‐resistant, ashless filter paper before instrumental analysis.

One reagent blank, one certified reference material, and at least one digestion duplicate were included in each digestion batch. A matrix‐appropriate certified reference material, such as NIST SRM 3232 Kelp Powder or an equivalent certified plant or algal reference material, was used to evaluate analytical recovery. Recovery was calculated as follows:

Recovery %=measured concentration÷certified concentration×100.

Relative percentage difference between digestion duplicates was calculated as follows:

RPD %=∣Duplicate 12−Duplicate ∣÷Duplicate 12+Duplicate ÷2×100.

All glassware and digestion vessels were soaked in 10% (v/v) nitric acid for at least 12 h and rinsed three times with ultrapure water before use. Only trace‐metal‐grade acids and ultrapure water with a resistivity of approximately 18.2 M*Ω*·cm were used for digestion, dilution, and preparation of blank and calibration standards.

#### 2.7.2. Instrumental Determination

Calcium, magnesium, potassium, sodium, iron, zinc, copper, and manganese were determined using a Shimadzu AA‐7000 atomic absorption spectrophotometer (Shimadzu Corporation, Kyoto, Japan) equipped with a flame‐atomization system. Element‐specific hollow‐cathode lamps were used, and measurements were conducted at the recommended analytical wavelengths: 422.7 nm for Ca, 285.2 nm for Mg, 766.5 nm for K, 589.0 nm for Na, 248.3 nm for Fe, 213.9 nm for Zn, 324.8 nm for Cu, and 279.5 nm for Mn. An air–acetylene flame was used, with gas flow and burner conditions optimized separately for each element according to the instrument manufacturer′s instructions.

To minimize chemical interference during calcium and magnesium determination, lanthanum chloride was added to the samples and calibration standards at the same final concentration. Samples containing mineral concentrations above the calibration range were diluted with an acidified ultrapure water solution before measurement. The corresponding dilution factors were incorporated into the final calculations.

Calibration curves were prepared from certified single‐element or multielement stock solutions using at least five concentration levels and a reagent blank. Calibration linearity was accepted when the coefficient of determination was at least 0.995. A reagent blank, continuing calibration verification standard, certified reference material, and analytical duplicate were included in each analytical batch. The analytical wavelengths, working calibration ranges, principal instrumental conditions, and prespecified quality‐control acceptance criteria are summarized in Table S2.

Selenium was determined using the graphite furnace configuration of the Shimadzu AA‐7000 instrument at an analytical wavelength of 196.0 nm. A palladium–magnesium matrix modifier was added to the standards and sample digests to reduce matrix interference during atomization. Selenium was quantified using matrix‐matched calibration standards and an optimized temperature program comprising drying, ashing, atomization, and cleaning stages. The analytical wavelength, working calibration range, matrix modifier, and general quality‐control acceptance criteria for selenium are summarized in Table S2.

Phosphorus was determined using the vanadomolybdate colorimetric method. An appropriate aliquot of each mineral digest was mixed with vanadate–molybdate reagent and allowed to develop color for 10 min at room temperature. Absorbance was measured at 420 nm using a UV–visible spectrophotometer against a reagent blank. Phosphorus concentration was calculated from a calibration curve prepared using certified phosphate standards treated under the same conditions as the samples.

#### 2.7.3. Analytical Quality Assurance

Instrument calibration was performed using a reagent blank and at least five concentration levels prepared from traceable certified standards. Calibration was accepted when *R* ^2^ ≥ 0.995. The prespecified acceptance criteria were 80%–120% recovery and a duplicate relative standard deviation (RSD) not exceeding 10%. Table S2 summarizes the instrumental conditions, working calibration ranges, and quality‐control acceptance criteria.

Limits of detection and quantification were calculated as follows:

LOD=3×SD of blank measurements÷calibration slope,

LOQ=10×SD of blank measurements÷calibration slope.

Results below the LOQ were reported as < LOQ and were not treated as quantified concentrations.

The following indices were calculated separately for each biological replicate:

Ca:Mg ratio=Ca÷Mg,

Na:K ratio=Na÷K,

Ca:P ratio=Ca÷P,

Zn:Cu ratio=Zn÷Cu,

Total macrominerals mggDW=Ca+Mg+K+Na+P.

Means and SDs were calculated from the three replicate‐level indices, not from ratios of the summarized species means.

Mineral measurements were obtained from three independently collected and separately processed biological samples per taxon. Calibration, blank, reference material, and duplicate quality‐control procedures were incorporated into the analytical workflow as described above. The relatively small dispersion of the Zn measurements among the three independent *C. glomerata* samples (4738 ± 96 *μ*g/g DW) indicates that the high concentration was consistently observed within the sampled biomass. However, the complete archived element‐specific quality‐control records required to retrospectively report the exact Zn recovery, duplicate precision, and independent confirmatory measurement are no longer available. Accordingly, the unusually high Zn concentration is reported conservatively as a sample‐specific analytical finding rather than as an established characteristic of *C. glomerata*. Independent confirmation using a second validated analytical procedure, together with analysis of the collection of water and potentially toxic elements, is warranted.

### 2.8. Determination of Selected Vitamins and Total Carotenoids

Thiamine, riboflavin, niacin, and total carotenoids were determined separately for each biological replicate using procedures adapted from Oser [20] and validated under the laboratory conditions. All extractions were performed under reduced light where appropriate.

For thiamine and riboflavin, approximately 2.0 g of dried sample was extracted with 50 mL of 0.1 M hydrochloric acid at 100°C for 30 min. After cooling and adjustment to pH 4.5, enzymatic hydrolysis was performed with takadiastase at 37°C for 16 h. Thiamine was oxidized to thiochrome and measured fluorometrically at excitation and emission wavelengths of 365 and 435 nm, respectively. Riboflavin was measured at excitation and emission wavelengths of 440 and 530 nm, respectively. Quantification was performed using certified thiamine and riboflavin standards.

Niacin was extracted from approximately 2.0 g of dried sample with 50 mL of 1.0 M sulfuric acid at 100°C for 30 min. After cooling, filtration, and pH adjustment, niacin was determined using the cyanogen–bromide–sulfanilic acid colorimetric procedure. Absorbance was measured at 450 nm, and concentrations were calculated using a certified nicotinic acid calibration standard. All cyanogen–bromide procedures were performed in a certified fume hood using appropriate protective equipment.

Total carotenoids were extracted from approximately 0.50 g of dried biomass using 25 mL of acetone–hexane (4:6, v/v). Extraction was repeated until the biomass was visually depleted of pigment. The pooled extract was brought to 50 mL, protected from light, and measured at 450 nm. Total carotenoids were quantified using an external *β*‐carotene calibration curve and expressed as milligrams of *β*‐carotene equivalents per 100 g DW.

Calibration curves were prepared using a reagent blank and at least five nonzero concentrations of the corresponding certified standard. Calibration was accepted when *R* ^2^ ≥ 0.995. The prespecified analytical acceptance criteria were 80%–120% recovery and a repeatability RSD not exceeding 10%. Samples producing responses outside the validated calibration range were diluted and reanalyzed. The extraction conditions, working calibration ranges, detection settings, and analytical acceptance criteria are summarized in Table S3.

### 2.9. Amino Acid Composition

Amino acid analysis was conducted using material obtained from the three biological samples of each algal taxon. However, the archived laboratory dataset retained for the present revision contained only the summarized taxon‐level amino acid profiles and did not contain the individual replicate‐level chromatographic results.

Approximately 100 mg of dried and homogenized sample was transferred to a hydrolysis tube and mixed with 5 mL of 6 M hydrochloric acid. The tubes were flushed with nitrogen, sealed, and heated at 110^°^C ± 2^°^C for 24 h. After hydrolysis, the samples were cooled, filtered, evaporated under reduced pressure at a temperature below 50°C, and reconstituted in 0.1 M hydrochloric acid. The reconstituted hydrolysates were passed through 0.22 *μ*m membrane filters before analysis.

Primary amino acids were derivatized before separation using *o*‐phthalaldehyde (OPA), whereas secondary amino acids were derivatized using 9‐fluorenylmethyl chloroformate (FMOC). Chromatographic separation was performed using an Agilent 1260 Infinity HPLC system (Agilent Technologies, Santa Clara, California, United States) equipped with an autosampler and fluorescence detector. An Agilent ZORBAX Eclipse AAA column (150 × 4.6 mm, 3.5 *μ*m) was maintained at 40°C. Mobile Phase A consisted of 40 mM sodium phosphate buffer adjusted to pH 7.8, and mobile Phase B consisted of acetonitrile–methanol–water (45:45:10, v/v/v). Separation was performed using gradient elution at a flow rate of 2.0 mL/min with an injection volume of 1 *μ*L. OPA derivatives were detected at excitation and emission wavelengths of 340 and 450 nm, respectively, whereas FMOC derivatives were detected at 266 and 305 nm, respectively. The complete gradient program is provided in Table S4.

Amino acids were identified by comparing their retention times with those of certified amino acid standards and quantified using external calibration curves prepared at a minimum of five concentration levels. Analytical performance was assessed using reagent blanks, duplicate hydrolyses, spiked samples, and calibration verification standards. Amino acid concentrations were converted to a protein basis as follows:

Amino acid mgg protein=amino acid mgg DW÷protein content gg DW.

The quantified amino acids included aspartic acid, glutamic acid, serine, glycine, histidine, arginine, threonine, alanine, proline, tyrosine, valine, isoleucine, leucine, phenylalanine, and lysine. Because acid hydrolysis converts asparagine and glutamine to aspartic and glutamic acids, respectively, the reported values represented Asp + Asn and Glu + Gln.

Methionine and cysteine were determined in separate portions after performic acid oxidation to methionine sulfone and cysteic acid, respectively, followed by acid hydrolysis and chromatographic analysis.

Because the individual replicate‐level amino acid results were unavailable, SDs could not be calculated or independently verified for the amino acid concentrations. The reported amino acid values therefore represent the archived summarized profiles and should not be interpreted as replicate‐supported estimates.

### 2.10. Protein‐Quality Indices

Indispensable amino acids were classified according to the FAO [21] adult reference pattern. The reference concentrations, expressed as milligrams per gram of protein, were histidine, 15; isoleucine, 30; leucine, 59; lysine, 45; sulfur‐containing amino acids (methionine + cysteine), 22; aromatic amino acids (phenylalanine + tyrosine), 38; threonine, 23; tryptophan, 6; and valine, 39.

Total measured essential amino acids (TEAAs) were calculated as the sum of the eight directly quantified indispensable amino acids included in the archived profiles:

TEAA mggprotein=histidine+isoleucine+leucine+lysine+methionine+phenylalanine+threonine+valine.

Cysteine and tyrosine were not added separately to TEAA because they are conditionally indispensable amino acids and were used only in the combined sulfur‐containing and aromatic amino acid scoring categories, respectively. Tryptophan was not quantified and was therefore excluded from TEAA.

For combined reference categories, the following equations were used:

Amino acid score=amino acid in test protein mgg protein÷corresponding FAO reference value mgg protein,

Sulfur amino acid score=methionine+cysteine÷22,

Aromatic amino acid score=phenylalanine+tyrosine÷38.

The chemical score was defined as the lowest calculated indispensable amino acid score, and the corresponding amino acid or combined amino acid category was identified as the first limiting amino acid.

The partial EAAI was calculated as the geometric mean of the eight available FAO‐relative scoring ratios:

EAAI=a1r1×a2r2×⋯×a8r818/,

where *a* is the concentration of an indispensable amino acid or combined scoring group in the test protein and *r* is its corresponding concentration in the FAO adult reference pattern. The eight scoring categories were histidine, isoleucine, leucine, lysine, methionine plus cysteine, phenylalanine plus tyrosine, threonine, and valine.

Because tryptophan was not measured, the EAAI and chemical score were considered partial protein‐quality indices. These indices cannot exclude tryptophan as the actual first limiting amino acid.

### 2.11. Statistical Scope and Software Version

Each independently collected biological sample was considered an experimental unit. Three independent biological samples were analyzed for each algal taxon (*n* = 3). Technical measurements obtained from the same biological sample were averaged before statistical analysis and were not treated as independent observations.

For variables for which replicate‐level biological data were available, including proximate composition, gross energy, selected vitamins, total carotenoids, mineral concentrations, and detergent‐fiber fractions, differences among the three sampled algal biomasses were evaluated using separate one‐way analyses of variance (ANOVA), with algal taxon as the fixed factor. When the omnibus ANOVA indicated a significant difference, Tukey′s honestly significant difference (HSD) test was used for pairwise comparisons. Statistical significance was established at *p* < 0.05.

Because only three independent biological samples were available per taxon, formal tests of normality and variance assumptions have limited diagnostic power. Inferential results were therefore interpreted cautiously together with the observed means, standard deviations, and biological replication rather than being used to infer universal species‐level differences.

Amino acid concentrations and derived protein‐quality indices were analyzed descriptively because the archived dataset contained only summarized taxon‐level amino acid profiles and not the individual replicate‐level chromatographic observations required for valid inferential testing. No inferential statistical comparisons were therefore made for these variables.

Ratios, sums, and calculated fractions were first determined separately for each available biological replicate, after which group means and SDs were calculated. Statistical analyses were performed using IBM SPSS Statistics Version 25.0 (IBM Corp., Armonk, New York, United States). A two‐sided significance level of *p* < 0.05 was used.

## 3. Results

### 3.1. Proximate Composition and Gross Energy

The three sampled algal biomasses showed distinct proximate composition patterns (Figure 1A,B). *M. cuaternaria* had the highest crude protein concentration (31.97 ± 0.41 g/100 g DW), followed by *G. chamissoi* (17.78 ± 0.32 g/100 g DW) and *C. glomerata* (11.03 ± 0.28 g/100 g DW). Crude fat followed the same order, with concentrations of 2.63 ± 0.08, 1.78 ± 0.07, and 0.63 ± 0.04 g/100 g DW, respectively. In contrast, *C. glomerata* contained the highest crude fiber (16.49 ± 0.33 g/100 g DW) and ash (32.09 ± 0.52 g/100 g DW) concentrations. The corresponding crude fiber and ash concentrations were 6.33 ± 0.16 and 10.20 ± 0.21 g/100 g DW in *M. cuaternaria* and 2.44 ± 0.11 and 14.40 ± 0.29 g/100 g DW in *G. chamissoi*.

**Figure 1:**
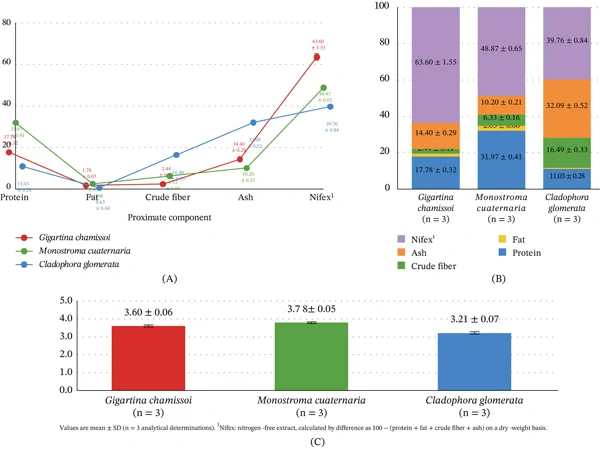
Proximate composition and gross energy of three edible algal biomasses collected in Peru. (A) Proximate composition: crude protein, crude fat, crude fiber, ash, and nitrogen‐free extract (Nifex), expressed as grams per 100 g dry weight (DW). (B) Proportional contribution of the measured proximate components to DW composition (% of total dry weight). (C) Gross energy expressed as kilocalories per gram DW. Values are the mean ± SD of three independent biological samples per taxon (*n* = 3). Error bars represent SD.

Nifex, calculated by difference, was highest in *G. chamissoi* (63.60 ± 1.55 g/100 g DW), intermediate in *M. cuaternaria* (48.87 ± 0.65 g/100 g DW), and lowest in *C. glomerata* (39.76 ± 0.84 g/100 g DW). The proportional profiles in Figure 1B therefore show that the sampled *G. chamissoi* biomass was characterized by a comparatively large Nifex fraction, *M. cuaternaria* had the greatest relative protein contribution, and *C. glomerata* had greater ash and crude fiber contributions.

Gross energy ranged from 3.21 to 3.78 kcal/g DW (Figure 1C). *M. cuaternaria* had the highest gross energy value (3.78 ± 0.05 kcal/g DW), followed by *G. chamissoi* (3.60 ± 0.06 kcal/g DW), whereas *C. glomerata* had the lowest value (3.21 ± 0.07 kcal/g DW).

One‐way ANOVA indicated differences among the three sampled biomasses for all proximate variables and gross energy (Table 1; all *p* < 0.001). Tukey′s HSD comparisons indicated pairwise differences for crude protein, crude fat, crude fiber, ash, and Nifex (all *p* < 0.001). For gross energy, the difference between *G. chamissoi* and *M. cuaternaria* was smaller but remained significant (*p* = 0.025), whereas both comparisons involving *C. glomerata* had *p* values below 0.001.

**Table 1:** One‐way ANOVA and Tukey′s HSD comparisons for proximate composition and gross energy.

Variable	ANOVA *F*(2, 6)	ANOVA *p*	*G* vs. *M*	*G* vs. *C*	*M* vs. *C*
Crude protein	2946.70	< 0.001	< 0.001	< 0.001	< 0.001
Crude fat	702.91	< 0.001	< 0.001	< 0.001	< 0.001
Crude fiber	3230.83	< 0.001	< 0.001	< 0.001	< 0.001
Ash	3047.22	< 0.001	< 0.001	< 0.001	< 0.001
Nifex^a^	368.91	< 0.001	< 0.001	< 0.001	< 0.001
Gross energy	69.46	< 0.001	0.025	< 0.001	< 0.001

*Note:* Statistical analyses were conducted using three independent biological observations per sampled taxon (*n* = 3). Technical readings obtained from the same biological sample were averaged before analysis and were not treated as independent observations. Separate one‐way ANOVAs were performed for each response variable, followed by Tukey′s honestly significant difference test. Statistical significance was established at *p* < 0.05.

Abbreviations: *C*, *C. glomerata*; *G*, *G. chamissoi*; *M*, *M. cuaternaria*.

^a^Nifex, nitrogen‐free extract calculated by difference.

### 3.2. Amino Acid Composition and Descriptive Protein‐Quality Indices

The amino acid profiles differed among the three sampled algal biomasses (Figure 2A). *M. cuaternaria* had the highest mean concentrations of all measured amino acids, including glutamic acid (96.3 mg/g protein), aspartic acid (72.8 mg/g protein), arginine (53.8 mg/g protein), alanine (48.2 mg/g protein), leucine (42.9 mg/g protein), and lysine (38.6 mg/g protein). The corresponding concentrations were generally intermediate in *G. chamissoi*, whereas *C. glomerata* had the lowest concentrations. Glutamic and aspartic acids were the most abundant measured amino acids in all three biomasses.

**Figure 2:**
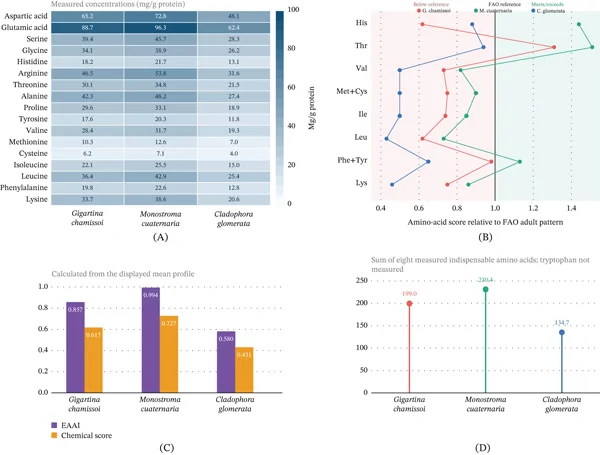
Amino acid composition, indispensable amino acid adequacy, and descriptive protein‐quality indices of three sampled algal biomasses. (A) Complete measured amino acid profile expressed as milligrams per gram of protein. Cell color intensity represents absolute concentration, whereas numerical labels show the corresponding mean values. (B) Indispensable amino acid scores calculated relative to the FAO [21] adult reference pattern. The vertical dashed line at 1.0 denotes equivalence to the reference concentration. Methionine plus cysteine and phenylalanine plus tyrosine were evaluated as combined sulfur‐containing and aromatic amino acid scoring groups, respectively. Open circles identify the lowest amino acid score for each taxon. (C) Protein‐quality indices. Essential amino acid index (EAAI) and chemical score calculated descriptively from the displayed mean amino acid profile. (D) Total concentration of the eight measured adult indispensable amino acids; tryptophan was not measured. Amino acid values and derived indices are descriptive taxon‐level summaries. Replicate‐level chromatographic observations were unavailable; therefore, SDs and inferential comparisons are not presented.

Evaluation against the FAO [21] adult reference pattern provided additional information on indispensable amino acid adequacy (Figure 2B). Histidine and threonine met or exceeded their respective reference concentrations in *G. chamissoi* and *M. cuaternaria*. The combined phenylalanine‐plus‐tyrosine score also exceeded 1.0 in *M. cuaternaria*. Leucine had the lowest amino acid score in all three samples, with calculated values of 0.617, 0.727, and 0.431 for *G. chamissoi*, *M. cuaternaria*, and *C. glomerata*, respectively. Leucine was therefore identified as the first limiting amino acid relative to the FAO adult reference pattern.

The EAAI values calculated descriptively from the displayed mean profiles were 0.857 for *G. chamissoi*, 0.994 for *M. cuaternaria*, and 0.580 for *C. glomerata* (Figure 2C). The corresponding chemical scores were 0.617, 0.727, and 0.431, respectively. The summed concentrations of the eight measured adult indispensable amino acids were 230.4 mg/g protein in *M. cuaternaria*, 199.0 mg/g protein in *G. chamissoi*, and 134.7 mg/g protein in *C. glomerata* (Figure 2D). Tryptophan was not quantified and was therefore excluded from these totals. Because the archived amino acid dataset contained only summarized taxon‐level profiles, the individual amino acid concentrations, EAAI, chemical score, and measured TEAA are presented descriptively without SDs or inferential comparisons.

### 3.3. Selected Vitamins and Total Carotenoids

The concentrations of selected vitamins and total carotenoids differed among the three sampled algal biomasses (Table 2). One‐way ANOVA indicated significant differences among taxa for thiamine (*F*(2, 6) = 19.62, *p* = 0.002), riboflavin (*F*(2, 6) = 21.00, *p* = 0.002), niacin (*F*(2, 6) = 1380.07, *p* < 0.001), and total carotenoids (*F*(2, 6) = 42.14, *p* < 0.001).

**Table 2:** Selected vitamin and total carotenoid concentrations in three sampled algal biomasses.

Parameter	Unit	*G. chamissoi*	*M. cuaternaria*	*C. glomerata*	*F*(2, 6)	*p* value
Thiamine (vitamin B1)	mg/100 g DW	0.130 ± 0.010^ab^	0.172 ± 0.020^a^	0.100 ± 0.010^b^	19.62	0.002
Riboflavin (vitamin B2)	mg/100 g DW	0.240 ± 0.020^a^	0.180 ± 0.020^b^	0.150 ± 0.010^b^	21.00	0.002
Niacin (vitamin B3)	mg/100 g DW	4.040 ± 0.180^a^	0.180 ± 0.020^b^	0.120 ± 0.010^b^	1380.07	< 0.001
Total carotenoids	mg/100 g DW	0.280 ± 0.030^b^	0.410 ± 0.030^a^	0.210 ± 0.020^c^	42.14	< 0.001

*Note:* Values are presented as mean ± SD (*n* = 3 independent biological samples per taxon). Within each row, means carrying different superscript letters differ significantly according to one‐way ANOVA followed by Tukey′s HSD test (*p* < 0.05). Means sharing the same superscript letter are not significantly different.

Abbreviation: DW, dry weight.

*M. cuaternaria* had the highest thiamine concentration (0.172 ± 0.020 mg/100 g DW), whereas *G. chamissoi* and *C. glomerata* contained 0.130 ± 0.010 and 0.100 ± 0.010 mg/100 g DW, respectively. Tukey′s HSD comparisons showed that *M. cuaternaria* differed significantly from both other biomasses, whereas the difference between *G. chamissoi* and *C. glomerata* was not significant.

Riboflavin was highest in *G. chamissoi* (0.240 ± 0.020 mg/100 g DW), which differed significantly from *M. cuaternaria* (0.180 ± 0.020 mg/100 g DW) and *C. glomerata* (0.150 ± 0.010 mg/100 g DW); the latter two did not differ significantly. A similar statistical pattern was observed for niacin, for which *G. chamissoi* had a markedly higher concentration (4.040 ± 0.180 mg/100 g DW) than *M. cuaternaria* (0.180 ± 0.020 mg/100 g DW) and *C. glomerata* (0.120 ± 0.010 mg/100 g DW).

Total carotenoid concentrations differed significantly among all three biomasses. *M. cuaternaria* had the highest concentration (0.410 ± 0.030 mg/100 g DW), followed by *G. chamissoi* (0.280 ± 0.030 mg/100 g DW) and *C. glomerata* (0.210 ± 0.020 mg/100 g DW).

### 3.4. Mineral Composition and Derived Mineral Indices

Mineral composition differed significantly among the three sampled algal biomasses (Table 3). One‐way ANOVA indicated significant differences among taxa for all measured mineral concentrations and derived mineral indices (*p* ≤ 0.004). *C. glomerata* had significantly higher concentrations of Ca, Mg, P, Fe, Cu, Mn, and Zn than the other sample biomasses. In contrast, *G. chamissoi* had the highest K and Na concentrations, whereas *M. cuaternaria* generally showed lower concentrations of the measured minerals.

**Table 3:** Mineral composition and derived mineral indices of three sampled algal biomasses.

Parameter	Unit	*G. chamissoi*	*M. cuaternaria*	*C. glomerata*	*F*(2, 6)	*p* value
Calcium (Ca)	mg/g DW	12.50 ± 0.38^b^	7.33 ± 0.26^c^	118.80 ± 2.74^a^	4615.26	< 0.001
Magnesium (Mg)	mg/g DW	10.20 ± 0.31^b^	4.12 ± 0.18^c^	109.00 ± 2.36^a^	5475.04	< 0.001
Potassium (K)	mg/g DW	38.06 ± 0.84^a^	6.22 ± 0.25^c^	18.45 ± 0.53^b^	2213.41	< 0.001
Sodium (Na)	mg/g DW	21.40 ± 0.61^a^	5.86 ± 0.22^c^	13.72 ± 0.44^b^	884.84	< 0.001
Phosphorus (P)	mg/g DW	3.82 ± 0.15^c^	4.73 ± 0.17^b^	5.91 ± 0.21^a^	103.49	< 0.001
Iron (Fe)	*μ*g/g DW	286 ± 11^b^	194 ± 9^c^	862 ± 28^a^	1196.45	< 0.001
Zinc (Zn)	*μ*g/g DW	84 ± 4^b^	52 ± 3^b^	4738 ± 96^a^	7080.29	< 0.001
Copper (Cu)	*μ*g/g DW	12.8 ± 0.7^b^	8.6 ± 0.5^c^	31.7 ± 1.3^a^	560.78	< 0.001
Manganese (Mn)	*μ*g/g DW	37.4 ± 1.8^b^	24.6 ± 1.2^c^	118.5 ± 4.7^a^	871.77	< 0.001
Selenium (Se)	*μ*g/g DW	0.62 ± 0.05^ab^	0.48 ± 0.04^b^	0.71 ± 0.06^a^	15.70	0.004
Ca:Mg ratio	Dimensionless	1.23 ± 0.05^b^	1.78 ± 0.10^a^	1.09 ± 0.03^b^	89.35	< 0.001
Na:K ratio	Dimensionless	0.56 ± 0.02^c^	0.94 ± 0.05^a^	0.74 ± 0.03^b^	85.58	< 0.001
Ca:P ratio	Dimensionless	3.27 ± 0.16^b^	1.55 ± 0.08^c^	20.10 ± 0.85^a^	1253.10	< 0.001
Zn:Cu ratio	Dimensionless	6.56 ± 0.48^b^	6.05 ± 0.50^b^	149.46 ± 6.84^a^	1300.74	< 0.001
Total measured macrominerals^a^	mg/g DW	85.98 ± 1.16^b^	28.26 ± 0.49^c^	265.88 ± 3.69^a^	9093.55	< 0.001

*Note:* Values are presented as mean ± SD on a dry weight basis (*n* = 3 independent biological samples per taxon). For each parameter, means with different superscript letters differ significantly according to one‐way ANOVA followed by Tukey′s HSD multiple‐comparison test (*p* < 0.05); means sharing at least one superscript letter are not significantly different. *F* statistics have df = 2, 6.

Abbreviation: DW, dry weight.

^a^Total measured macrominerals = Ca + Mg + K + Na + P.

The most pronounced difference was observed for Zn (*F*(2, 6) = 7080.29, *p* < 0.001). *C. glomerata* contained 4738 ± 96 *μ*g/g DW, significantly exceeding the concentrations measured in *G. chamissoi* (84 ± 4 *μ*g/g DW) and *M. cuaternaria* (52 ± 3 *μ*g/g DW). Tukey′s HSD test did not indicate a significant difference between the latter two biomasses. Given the unusually high magnitude of the Zn concentration in *C. glomerata*, this result is interpreted as a sample‐specific analytical observation and not as a typical species‐level concentration; the analytical validation limitations and need for independent confirmation are considered separately below.

Significant differences were also observed in the derived mineral indices. *M. cuaternaria* had the highest Ca and Na ratios, whereas *C. glomerata* had the highest Ca and Zn ratios. Total measured macrominerals differed significantly among all three biomasses and were highest in *C. glomerata* (265.88 ± 3.69 mg/g DW), followed by *G. chamissoi* (85.98 ± 1.16 mg/g DW) and *M. cuaternaria* (28.26 ± 0.49 mg/g DW).

The Zn concentration measured in *C. glomerata* (4738 ± 96 *μ*g/g DW) was markedly higher than those measured in *G. chamissoi* and *M. cuaternaria*. This unusually high value is reported as an analytical observation specific to the sampled biomass and should not be interpreted as a typical species‐level Zn concentration. Freshwater *Cladophora* spp. can accumulate dissolved metals through surface adsorption, ion exchange, and intracellular sequestration, and metal accumulation can vary substantially with local water chemistry and exposure conditions. Nevertheless, because contemporaneous water chemistry measurements and complete element‐specific validation records for retrospective independent verification were unavailable, the magnitude of the Zn concentration cannot be attributed definitively to environmental accumulation alone. The result is therefore treated cautiously as a sample‐specific finding requiring independent analytical confirmation.

#### 3.4.1. Dietary Zinc Intake Context

The nutritional relevance of the high Zn concentration measured in *C. glomerata* was examined using a portion‐based intake scenario (Figure 3). At the measured concentration of 4.738 ± 0.096 mg/g DW, dried portions of 1, 3, and 5 g would contain approximately 4.74, 14.21, and 23.69 mg total Zn, respectively. The EFSA adult average requirement range of 6.2–12.7 mg/day, which varies by sex and dietary phytate intake, would be reached by approximately 1.31–2.68 g DW. The adult tolerable upper intake level of 25 mg/day would be reached at approximately 5.28 g DW (EFSA NDA Panel, 2014).

**Figure 3:**
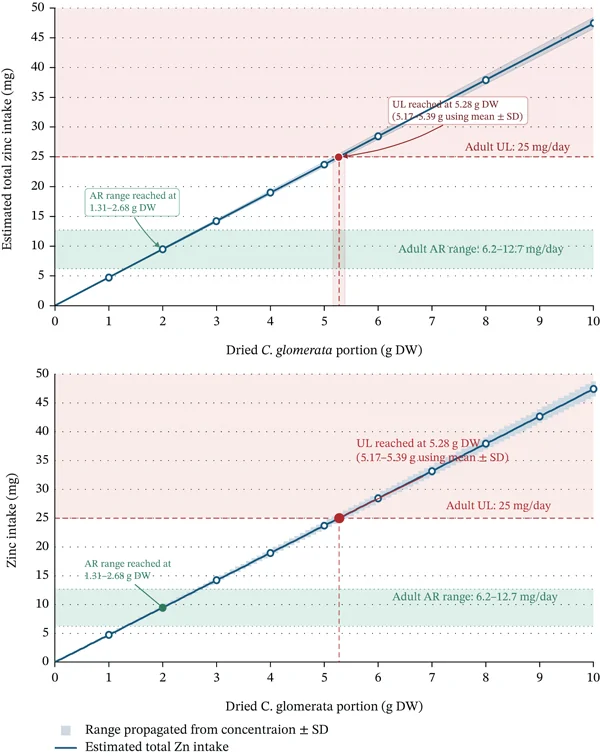
Dietary zinc intake context for dried *Cladophora glomerata*. The total Zn intake was calculated from the measured concentration of 4.738 ± 0.096 mg/g DW. The blue line represents intake calculated using the mean concentration, whereas the surrounding band propagates the reported concentration SD across portion sizes. The green region represents the EFSA adult average requirement range of 6.2–12.7 mg/day, which varies according to sex and dietary phytate intake. The red dashed line represents the adult tolerable upper intake level of 25 mg/day. Based on the measured concentration, this level would be reached by approximately 5.28 g DW, with a concentration‐based range of 5.17–5.39 g. This screening calculation represents total Zn and does not account for bioaccessibility, processing losses, background dietary exposure, or variation beyond the analyzed biomass.

### 3.5. Fiber Fractions and Derived Cell Wall Characteristics

Fiber composition and derived cell wall characteristics differed among the three sampled algal biomasses (Table 4). One‐way ANOVA indicated significant differences among taxa for all evaluated fiber variables and derived ratios (*p* ≤ 0.016).

**Table 4:** Fiber fractions and derived cell wall characteristics of three sampled algal biomasses.

Parameter	Unit	*G. chamissoi*	*M. cuaternaria*	*C. glomerata*	*F*(2, 6)	*p* value
Neutral detergent fiber (NDF)	g/100 g DW	14.40 ± 0.43^c^	60.20 ± 1.28^a^	38.60 ± 0.91^b^	1781.99	< 0.001
Acid detergent fiber (ADF)	g/100 g DW	3.80 ± 0.18^c^	8.23 ± 0.34^b^	27.90 ± 0.72^a^	2222.41	< 0.001
Hemicellulose	g/100 g DW	10.60 ± 0.47^b^	51.97 ± 1.32^a^	10.70 ± 1.16^b^	1547.96	< 0.001
Cellulose	g/100 g DW	3.07 ± 0.19^c^	5.71 ± 0.36^b^	23.74 ± 0.75^a^	1564.06	< 0.001
Lignin	g/100 g DW	0.73 ± 0.06^c^	2.52 ± 0.13^b^	4.16 ± 0.22^a^	384.44	< 0.001
NDF:ADF ratio	Dimensionless	3.79 ± 0.21^b^	7.31 ± 0.34^a^	1.38 ± 0.05^c^	493.50	< 0.001
Hemicellulose:NDF ratio	Dimensionless	0.74 ± 0.04^b^	0.86 ± 0.03^a^	0.28 ± 0.03^c^	248.12	< 0.001
Cellulose:ADF ratio	Dimensionless	0.81 ± 0.06^ab^	0.69 ± 0.05^b^	0.85 ± 0.03^a^	8.91	0.016
Lignin:ADF ratio	Dimensionless	0.19 ± 0.02^b^	0.31 ± 0.02^a^	0.15 ± 0.01^b^	69.33	< 0.001
Structural carbohydrate^a^	g/100 g DW	13.67 ± 0.50^c^	57.68 ± 1.37^a^	34.44 ± 1.38^b^	1082.17	< 0.001

*Note:* Values are presented as mean ± SD on a dry weight basis (*n* = 3 independent biological samples per taxon). For each parameter, means with different superscript letters differ significantly according to one‐way ANOVA followed by Tukey′s HSD multiple‐comparison test (*p* < 0.05); means sharing at least one superscript letter are not significantly different. *F* statistics have df = 2, 6.

Abbreviation: DW, dry weight.

^a^Structural carbohydrate was calculated as hemicellulose + cellulose.

*M. cuaternaria* had the highest NDF concentration (60.20 ± 1.28 g/100 g DW), significantly exceeding *C. glomerata* (38.60 ± 0.91 g/100 g DW) and *G. chamissoi* (14.40 ± 0.43 g/100 g DW). Hemicellulose was also substantially higher in *M. cuaternaria* (51.97 ± 1.32 g/100 g DW), whereas *G. chamissoi* (10.60 ± 0.47 g/100 g DW) and *C. glomerata* (10.70 ± 1.16 g/100 g DW) did not differ significantly from each other.

In contrast, *C. glomerata* had the highest ADF (27.90 ± 0.72 g/100 g DW), cellulose (23.74 ± 0.75 g/100 g DW), and lignin (4.16 ± 0.22 g/100 g DW) concentrations, with significant differences among all three sampled biomasses for these variables. The NDF and hemicellulose ratios were highest in *M. cuaternaria*, whereas *C. glomerata* had the highest cellulose ratio. For the latter variable, the overall ANOVA was significant (*F*(2, 6) = 8.91, *p* = 0.016), although not all Tukey pairwise comparisons were significant.

Structural carbohydrate, calculated as the sum of hemicellulose and cellulose, differed significantly among all three biomasses (*F*(2, 6) = 1082.17, *p* < 0.001) and was highest in *M. cuaternaria* (57.68 ± 1.37 g/100 g DW), followed by *C. glomerata* (34.44 ± 1.38 g/100 g DW) and *G. chamissoi* (13.67 ± 0.50 g/100 g DW).

## 4. Discussion

The present study provides a comparative description of the proximate composition, selected vitamins, carotenoids, minerals, detergent‐fiber fractions, and amino acid profiles of three edible algal biomasses collected from contrasting Peruvian aquatic environments. The sampled biomasses differed substantially in their measured nutritional characteristics: *M. cuaternaria* was distinguished by higher crude protein and measured indispensable amino acid concentrations, *G. chamissoi* by higher Nifex and niacin, and *C. glomerata* by higher ash, structural fiber, Ca, Mg, and Zn concentrations. These differences should be interpreted as characteristics of the samples collected under the defined geographic and temporal conditions rather than as universal species‐level profiles, because each taxon was collected from a different water body during a single sampling period.

The protein concentration of *M. cuaternaria* (31.97 g/100 g DW) was higher than the values reported for several previously characterized edible green algae. Yaich et al. [10], for example, reported approximately 8.5% protein in *U. lactuca*, whereas Ortiz et al. [9] reported protein concentrations of approximately 10.8%–13.7% in the edible algae *Codium fragile*, *Gracilaria chilensis*, and *Macrocystis pyrifera*. The protein concentration of *C. glomerata* in the present study (11.03 g/100 g DW) was closer to this previously reported range. The comparatively high protein concentration measured in *M. cuaternaria* is consistent with the broad protein variability recognized among green macroalgae, although differences in habitat, nitrogen availability, season, drying, and the nitrogen‐to‐protein conversion factor can strongly influence reported values [3, 16]. The conventional factor of 6.25 used in the present study may also overestimate true protein if the biomass contains substantial nonprotein nitrogen; consequently, the values should be interpreted as crude protein estimates.

The measured amino acid profiles provided a more detailed evaluation than protein concentration alone. *M. cuaternaria* had the highest concentrations of the measured amino acids and the greatest summed concentration of the eight quantified adult indispensable amino acids (230.4 mg/g protein), followed by *G. chamissoi* (199.0 mg/g protein) and *C. glomerata* (134.7 mg/g protein). Previous analyses of edible algae have similarly demonstrated substantial variation in indispensable amino acid composition among taxa and morphological structures [8, 16]. Importantly, comparison with the FAO [21] adult reference pattern showed that leucine, rather than lysine, had the lowest relative score in all three sampled biomasses. The calculated leucine scores were 0.617, 0.727, and 0.431 for *G. chamissoi*, *M. cuaternaria*, and *C. glomerata*, respectively. The descriptive EAAI was therefore highest in *M. cuaternaria* (0.994), followed by *G. chamissoi* (0.857) and *C. glomerata* (0.580). These indices permit comparison of amino acid balance against a nutritional reference, but they do not measure protein digestibility or bioavailability. Tryptophan was not quantified, and the reported indices should therefore be interpreted as assessments based on the amino acids included in the analysis.

The proximate composition of *G. chamissoi* was characterized by a comparatively high Nifex concentration of 63.60 g/100 g DW. This value was broadly consistent with the carbohydrate‐rich composition reported for several red macroalgae. Arakaki and Ramos [13] documented substantial variation in protein, carbohydrate, lipid, and ash concentrations among edible Peruvian seaweeds and reported carbohydrate‐rich profiles in several red algal groups. Ortiz et al. [9] similarly reported calculated carbohydrate concentrations of approximately 66%–75% in selected Chilean macroalgae. Nevertheless, Nifex is a residual value calculated by difference and includes cumulative analytical error. It cannot be interpreted as a direct measurement of soluble carbohydrate, carrageenan, or another specific polysaccharide. Although sulfated galactans and carrageenan‐type polymers are characteristic of several red algae [5, 14, 15], their concentration and structure would require direct extraction and chemical characterization.

The comparatively high crude fiber and ash concentrations in *C. glomerata* were consistent with the structural characteristics of filamentous freshwater algae. Its ash concentration of 32.09 g/100 g DW exceeded the 19.6% reported for *U. lactuca* by Yaich et al. [10] but remained within the broad mineral variability described for macroalgal biomasses [2, 3]. The Van Soest results further distinguished the cell wall characteristics of the sampled materials. *M. cuaternaria* contained the highest NDF (60.20 g/100 g DW) and hemicellulose (51.97 g/100 g DW), whereas *C. glomerata* contained the highest ADF (27.90 g/100 g DW) and cellulose (23.74 g/100 g DW). The NDF concentration in *M. cuaternaria* was similar in magnitude to the 54.0% fiber reported for *U. lactuca* by Yaich et al. [10] and the 60.5% total dietary fiber reported by Ortiz et al. [8]. These comparisons are contextual rather than direct because total dietary fiber and Van Soest NDF are operationally different measurements. Furthermore, detergent‐fiber procedures were originally developed for terrestrial forage and require careful ash correction when applied to mineral‐rich algal matrices.

The vitamin results also indicated contrasting compositional patterns. *M. cuaternaria* contained the highest thiamine and total carotenoid concentrations, whereas *G. chamissoi* contained the highest riboflavin and niacin concentrations. The high carotenoid value relative to the other two sampled biomasses is consistent with the photosynthetic pigment composition expected in green algae, although carotenoid concentrations vary with irradiance, nutrient availability, maturity, extraction solvent, drying, and storage. Ortiz et al. [8] and Ortiz et al. [9] also reported taxon‐dependent differences in pigments and vitamins among edible algae. The higher niacin concentration in *G. chamissoi* (4.04 mg/100 g DW) was consistent with the general observation that some edible red algae can provide B‐complex vitamins [22]. However, differences among studies should be interpreted cautiously because vitamin extraction, vitamer specificity, degradation during drying, and analytical detection methods can produce substantial variation. The present vitamin results therefore characterize the analyzed biomasses but do not establish their contribution to dietary requirements without information on serving size, retention after processing, and bioavailability.

The mineral results showed the most pronounced compositional contrast. *C. glomerata* contained 118.80 mg/g Ca, 109.00 mg/g Mg, and 4738 *μ*g/g Zn, together with higher Fe, Cu, Mn, and Se concentrations than the other two sampled biomasses. This pattern agrees with the recognized capacity of freshwater filamentous algae to accumulate dissolved elements through cell wall binding, ion exchange, surface adsorption, and intracellular sequestration [23–25]. Hydroxyl, carboxyl, and other functional groups associated with algal cell walls can bind metal ions, although accumulation depends strongly on water chemistry, pH, competing ions, exposure history, and physiological state [6, 25]. The mineral differences observed here therefore cannot be attributed solely to taxonomy because the three taxa were collected from environmentally distinct water bodies.

The exceptionally high Zn concentration measured in *C. glomerata* (4738 ± 96 *μ*g/g DW; 4.738 ± 0.096 mg/g DW) warrants caution. The observation was consistent across the three independently collected biological samples, as reflected by the relatively small between‐sample SD, but its magnitude is unusual and should not be generalized to *C. glomerata* as a species. Freshwater filamentous algae can accumulate metals through adsorption to cell wall functional groups, ion exchange, and intracellular sequestration, and the extent of accumulation depends strongly on local water chemistry and exposure history. Because contemporaneous measurements of Zn in the collection water and complete, archived element‐specific validation records were unavailable, environmental accumulation cannot be conclusively distinguished from other potential variation, and independent analytical confirmation is required [26, 27].

At the measured concentration, a 5‐g portion of dried biomass would contain approximately 23.69 mg total Zn, whereas approximately 5.28 g would contain 25 mg Zn, corresponding to the adult tolerable upper intake level used in the EFSA dietary reference evaluation. These calculations demonstrate that the high Zn concentration should not be interpreted automatically as a nutritional advantage. Total concentration does not indicate the fraction released during digestion or absorbed by the body, and the calculation does not account for background dietary Zn intake. Moreover, mechanisms that concentrate Zn may also promote accumulation of potentially toxic elements. Because As, Cd, Pb, Hg, and inorganic arsenic were not quantified, the present results are insufficient to establish the food safety of the sampled *C. glomerata*. Independent confirmation of Zn, contaminant screening, mineral bioaccessibility assessment, and portion‐specific exposure evaluation are therefore required before this biomass can be considered for food or mineral fortification applications.

The FAO‐relative amino acid visualization and the Zn portion‐size analysis were used to improve interpretation of the measured composition. The amino acid visualization expresses the quantified amino acid categories relative to the FAO adult reference pattern, whereas the Zn analysis translates the measured concentration into a portion‐based intake scenario. These visualizations summarize the underlying measurements and do not constitute independent evidence or establish causal relationships [28].

Antioxidant activity and other bioactive compounds were not assessed in the present study. This omission limits the functional characterization of the samples because algae may contain phenolic compounds, pigments, polysaccharides, and other metabolites associated with antioxidant activity [2, 29]. However, DPPH, ABTS, and FRAP results are highly dependent on extraction solvent, extraction time, extract concentration, reaction conditions, and calibration standard. Literature‐derived antioxidant values were therefore not combined with the present experimental dataset. Future studies should determine total phenolics, individual pigments, and antioxidant capacity using standardized extraction and reporting procedures that permit meaningful comparison among samples.

### 4.1. Study Limitations

Several limitations affect the interpretation and generalizability of the findings. First, each taxon was collected from a different water body during one sampling period; therefore, taxon, location, habitat, and environmental conditions were confounded. The observed differences cannot be assigned exclusively to species identity. Second, only three independent biological samples were analyzed per taxon, limiting statistical precision and the reliability of distributional assumptions. Third, some derived amino acid indices were calculated descriptively from the displayed mean profiles because replicate‐level amino acid observations were unavailable for recalculation. These indices were therefore not subjected to inferential testing. Fourth, mineral bioaccessibility, protein digestibility, antinutritional factors, iodine, and potentially toxic elements were not measured. Consequently, nutritional availability and food safety cannot be established from total concentrations alone. Fifth, antioxidant activity, phenolic compounds, sensory acceptability, and the effects of food processing were outside the scope of the study.

Future investigations should include repeated sampling across locations and seasons, larger numbers of independent biological samples, and environmental measurements such as water chemistry and dissolved element concentrations. Replicate‐level amino acid indices, in vitro protein digestibility, mineral bioaccessibility, iodine and contaminant screening, antioxidant activity, and processing stability should also be evaluated. Such studies would help distinguish taxonomic effects from environmental variation and determine whether the nutritional characteristics observed here remain consistent across harvesting conditions and food‐processing systems.

### 4.2. Scientific and Practical Implications

The findings provide a comparative nutritional dataset for three edible algal biomasses collected from distinct Peruvian aquatic environments. The study demonstrates that nutritional value should be evaluated at the level of individual algal materials rather than generalized across “seaweeds” or freshwater algae. The sampled *M. cuaternaria* biomass was distinguished by its comparatively high crude protein concentration, measured indispensable amino acid total, and amino acid balance relative to the FAO adult reference pattern. The sampled *G. chamissoi* biomass contained the highest Nifex and niacin concentrations, whereas *C. glomerata* contained comparatively high structural fiber and mineral concentrations. These complementary characteristics provide a basis for selecting algal materials for more focused food composition, processing, and nutritional bioavailability studies [30, 31].

From an academic perspective, the amino acid adequacy framework provides more meaningful information than protein concentration or a single amino acid alone. Converting measured amino acid concentrations to FAO‐relative scores identified leucine as the first limiting amino acid in all three biomasses. This finding demonstrates that a comparatively high protein concentration does not necessarily indicate complete amino acid adequacy. Similarly, the Zn intake context analysis demonstrates how concentration data can be translated into a portion‐based exposure scenario. These analytical transformations improve interpretation while avoiding unsupported conclusions from complex multivariate models.

The results also have implications for product development. *M. cuaternaria* may warrant further evaluation as a protein‐containing ingredient, but protein digestibility, amino acid bioavailability, sensory characteristics, and processing stability must first be established. *G. chamissoi* may be relevant for carbohydrate‐ and hydrocolloid‐oriented applications; however, Nifex is not a direct measure of carrageenan or soluble polysaccharides, and targeted structural analyses are required. The fiber composition of *C. glomerata* suggests possible technological functionality, but its exceptionally high Zn concentration prevents an immediate recommendation for food or fortification applications.

The mineral results are particularly important for food safety evaluation. Although the high Ca, Mg, Fe, and Zn concentrations in *C. glomerata* may initially suggest micronutrient potential, total mineral concentration alone cannot establish nutritional benefit. Mineral bioaccessibility, processing losses, background dietary intake, and coaccumulation of potentially toxic elements must be considered. Therefore, future evaluation of *C. glomerata* should prioritize analytical confirmation of Zn, measurement of As, Cd, Pb, Hg, and iodine, and assessment of mineral release under gastrointestinal conditions.

At the community and food system level, locally available algae could contribute to dietary diversification and the development of regionally adapted food products. Nevertheless, their use should be supported by standardized harvesting, species authentication, environmental monitoring, contaminant control, and serving size guidance. The present findings should therefore be regarded as a screening foundation for subsequent nutritional and food safety studies rather than as evidence supporting immediate dietary intervention.

### 4.3. Study Limitations

The study was limited by the absence of deposited voucher specimens, precise sampling coordinates, contemporaneous water‐quality measurements, and complete element‐specific validation records. These omissions restrict independent verification of species identity, collection conditions, and analytical accuracy, particularly for the unusually high Zn concentration in *C. glomerata*. In addition, only summarized taxon‐level amino acid profiles were available; therefore, variability in amino acid concentrations and derived indices could not be calculated, and these results were interpreted descriptively without inferential comparisons. Future studies should deposit voucher specimens, document complete sampling and environmental metadata, confirm unusually high mineral concentrations independently, and publicly archive replicate‐level chromatograms, calibration records, quality‐control results, and calculation files.

## 5. Conclusion

This study comparatively characterized the proximate composition, gross energy, selected vitamins, total carotenoids, minerals, detergent‐fiber fractions, and amino acid profiles of *G. chamissoi*, *M. cuaternaria*, and *C. glomerata* collected from three Peruvian water bodies. Within the analyzed biomasses, *M. cuaternaria* had the highest crude protein concentration and the greatest measured indispensable amino acid total, whereas its FAO‐relative profile produced the highest descriptive EAAI. *G. chamissoi* had the highest Nifex and niacin concentrations, whereas *C. glomerata* contained the highest ash, ADF, cellulose, Ca, Mg, and Zn concentrations.

The amino acid adequacy analysis identified leucine as the first limiting amino acid in all three biomasses, demonstrating that protein concentration alone was insufficient to describe protein quality. The portion‐based Zn analysis further showed that approximately 5.28 g of dried *C. glomerata* at the measured concentration would contain 25 mg total Zn. Consequently, the high mineral concentration should not be interpreted solely as a nutritional advantage, and the biomass cannot be recommended for food or fortification use without analytical confirmation, bioaccessibility testing, and contaminant screening.

Because each taxon was collected from a different location during one sampling period and only three independent biological samples were examined per taxon, the findings describe the sampled biomasses rather than universal species‐level nutritional characteristics. Further multisite and multiseason studies should incorporate larger numbers of biological samples, protein digestibility, mineral bioaccessibility, iodine and toxic element screening, antioxidant and bioactive compound analysis, sensory evaluation, and processing stability. These steps are necessary to determine whether the observed nutritional characteristics can support safe and reproducible food applications.

## Funding

No funding was received for this manuscript.

## Ethics Statement

The authors have nothing to report.

## Conflicts of Interest

The authors declare no conflicts of interest.

## Supporting information


**Supporting Information** Additional supporting information can be found online in the Supporting Information section. Table S1: The sampling design and collection metadata. Table S2: The analytical conditions for mineral determination. Table S3: The analytical methods for vitamins and carotenoids. Table S4: The chromatographic conditions for amino acid analysis. Figure S1: The representative appearance of the three studied algal species.

## Data Availability

The data that support the findings of this study are available on request from the corresponding author. The data are not publicly available due to privacy or ethical restrictions.
