# Supplementary material for: Comparative Nutritional Profiles of Three Edible Algae Collected From Peruvian Marine and Freshwater Environments
Source: Int J Food Sci. 2026 Nov 14;2026:2170707. doi: 10.1155/ijfo/2170707 (PMC13769043; doi:10.1155/ijfo/2170707)
Supplement: Supplementary file 1 [file IJFO-2026-2170707-s001.docx]

**Supplementary materials**

**Table S1.** Sampling design and available collection metadata for the three studied algal biomasses.

| **Taxon** | **Collection area** | **Environment** | **Sampling period** | **Fresh mass per biological replicate** | **Replication and separation** | **Collection procedure** | **Transport and processing** | **Voucher status** |
| --- | --- | --- | --- | --- | --- | --- | --- | --- |
| *Chondracanthus chamissoi* (syn. *Gigartina chamissoi*) | Coastal waters near Ancón, Lima, Peru | Marine coastal environment | May–August 2024 | Approximately 300 g | Three independent biological samples (G1–G3) collected from separate thalli or patches located at least 10 m apart | Manual collection during low tide or shallow submersion using clean equipment | Samples transported separately at approximately 4 ± 2 °C and processed within 12 h | Not deposited; accession number unavailable |
| *Monostroma quaternarium* | Lake Titicaca, Puno, Peru | Freshwater lake | May–August 2024 | Approximately 300 g | Three independent biological samples (M1–M3) collected from separate thalli or patches located at least 10 m apart | Manual collection from accessible submerged substrates using clean equipment | Samples transported separately at approximately 4 ± 2 °C and processed within 12 h | Not deposited; accession number unavailable |
| *Cladophora glomerata* | Urubamba River, Cusco, Peru | Freshwater river | May–August 2024 | Approximately 300 g | Three independent biological samples (C1–C3) collected from separate algal patches located at least 10 m apart | Manual collection from accessible submerged substrates using clean equipment | Samples transported separately at approximately 4 ± 2 °C and processed within 12 h | Not deposited; accession number unavailable |

Exact collection dates, geographic coordinates, site-specific elevations or collection depths, contemporaneous water-quality measurements, and voucher accession numbers were unavailable. These omissions limit independent verification of collection-site conditions and species identity

**Table S2.** Instrumental conditions, working calibration ranges, and prespecified analytical acceptance criteria for mineral determination.

| **Element** | **Technique** | **Wavelength (nm)** | **Working calibration range** | **Principal analytical condition** | **Reporting unit** | **Calibration criterion** | **Recovery criterion** | **Precision criterion** |
| --- | --- | --- | --- | --- | --- | --- | --- | --- |
| Calcium (Ca) | Flame AAS | 422.7 | 0.0–5.0 mg/L | Air–acetylene flame; lanthanum chloride added to samples and standards | mg/g DW | R² ≥ 0.995 | 80–120% | Duplicate RSD ≤10% |
| Magnesium (Mg) | Flame AAS | 285.2 | 0.0–1.0 mg/L | Air–acetylene flame; lanthanum chloride added to samples and standards | mg/g DW | R² ≥ 0.995 | 80–120% | Duplicate RSD ≤10% |
| Potassium (K) | Flame AAS | 766.5 | 0.0–2.0 mg/L | Air–acetylene flame; additional sample dilution when required | mg/g DW | R² ≥ 0.995 | 80–120% | Duplicate RSD ≤10% |
| Sodium (Na) | Flame AAS | 589.0 | 0.0–1.0 mg/L | Air–acetylene flame; additional sample dilution when required | mg/g DW | R² ≥ 0.995 | 80–120% | Duplicate RSD ≤10% |
| Iron (Fe) | Flame AAS | 248.3 | 0.0–5.0 mg/L | Air–acetylene flame | µg/g DW | R² ≥ 0.995 | 80–120% | Duplicate RSD ≤10% |
| Zinc (Zn) | Flame AAS | 213.9 | 0.0–2.0 mg/L | Air–acetylene flame; additional dilution applied to high-concentration samples | µg/g DW | R² ≥ 0.995 | 80–120% | Duplicate RSD ≤10% |
| Copper (Cu) | Flame AAS | 324.8 | 0.0–5.0 mg/L | Air–acetylene flame | µg/g DW | R² ≥ 0.995 | 80–120% | Duplicate RSD ≤10% |
| Manganese (Mn) | Flame AAS | 279.5 | 0.0–2.0 mg/L | Air–acetylene flame | µg/g DW | R² ≥ 0.995 | 80–120% | Duplicate RSD ≤10% |
| Selenium (Se) | Graphite-furnace AAS | 196.0 | 0–50 µg/L | Palladium–magnesium matrix modifier | µg/g DW | R² ≥ 0.995 | 80–120% | Duplicate RSD ≤10% |
| Phosphorus (P) | Vanadomolybdate UV–Vis method | 420 | 0.0–10.0 mg/L | Color development for 10 min at room temperature | mg/g DW | R² ≥ 0.995 | 80–120% | Duplicate RSD ≤10% |

**Notes:** AAS, atomic absorption spectrophotometry; DW, dry weight; RSD, relative standard deviation; UV–Vis, ultraviolet–visible spectrophotometry. Calibration curves were prepared using a reagent blank and at least five non-zero concentration levels. Samples producing signals above the working calibration range were diluted and reanalyzed. The dilution factor was included in the final concentration calculation. Recovery and precision values shown in this table are prespecified acceptance criteria and must not be interpreted as observed analytical results.

Limits of detection and quantification were calculated separately for each element as follows: LOD = 3 × SD of blank measurements ÷ calibration slope; LOQ = 10 × SD of blank measurements ÷ calibration slope. Results below the LOQ were reported as <LOQ and were not treated as quantified concentrations. Element-specific observed LODs, LOQs, recoveries, and duplicate RSDs were unavailable for inclusion in the supplementary dataset.

**Table S3.** Analytical conditions, working calibration ranges, and prespecified acceptance criteria for selected vitamins and total carotenoids.

| **Analyte** | **Sample mass** | **Extraction and hydrolysis** | **Detection condition** | **Calibration standard** | **Working calibration range** | **Reporting unit** | **Calibration criterion** | **Recovery criterion** | **Repeatability criterion** |
| --- | --- | --- | --- | --- | --- | --- | --- | --- | --- |
| Thiamine (vitamin B1) | 2.0 g | 50 mL of 0.1 M HCl at 100 °C for 30 min; adjusted to pH 4.5; takadiastase hydrolysis at 37 °C for 16 h | Thiochrome fluorescence: excitation 365 nm and emission 435 nm | Certified thiamine hydrochloride | 0.01–0.20 mg/L | mg/100 g DW | R² ≥ 0.995 | 80–120% | RSD ≤10% |
| Riboflavin (vitamin B2) | 2.0 g | 50 mL of 0.1 M HCl at 100 °C for 30 min; adjusted to pH 4.5; takadiastase hydrolysis at 37 °C for 16 h | Fluorescence: excitation 440 nm and emission 530 nm | Certified riboflavin | 0.01–0.20 mg/L | mg/100 g DW | R² ≥ 0.995 | 80–120% | RSD ≤10% |
| Niacin (vitamin B3) | 2.0 g | 50 mL of 1.0 M H₂SO₄ at 100 °C for 30 min; cooled, filtered, and adjusted to the required reaction pH | Cyanogen-bromide–sulfanilic-acid colorimetry at 450 nm | Certified nicotinic acid | 0.10–5.00 mg/L | mg/100 g DW | R² ≥ 0.995 | 80–120% | RSD ≤10% |
| Total carotenoids | 0.50 g | Repeated extraction with acetone–hexane (4:6, v/v); pooled extract adjusted to 50 mL | UV–Vis absorbance at 450 nm | β-Carotene | 0.5–10.0 µg/mL | mg β-carotene equivalents/100 g DW | R² ≥ 0.995 | 80–120% | RSD ≤10% |

DW, dry weight; RSD, relative standard deviation; UV–Vis, ultraviolet–visible spectrophotometry. Recovery and repeatability values are prespecified acceptance criteria and should not be interpreted as observed validation results.

**Table S4.** Chromatographic conditions and gradient program for OPA/FMOC amino-acid determination.

| **Parameter** | **Analytical condition** |
| --- | --- |
| HPLC system | Agilent 1260 Infinity HPLC system |
| Autosampler | Automated pre-column derivatization configuration |
| Detector | Fluorescence detector |
| Column | Agilent ZORBAX Eclipse AAA, 150 × 4.6 mm, 3.5 µm |
| Column temperature | 40 °C |
| Mobile phase A | 40 mM sodium phosphate buffer, pH 7.8 |
| Mobile phase B | Acetonitrile–methanol–water (45:45:10, v/v/v) |
| Flow rate | 2.0 mL/min |
| Injection volume | 1 µL |
| Primary amino-acid derivatization | OPA |
| Secondary amino-acid derivatization | FMOC |
| OPA detection | Excitation 340 nm; emission 450 nm |
| FMOC detection | Excitation 266 nm; emission 305 nm |
| Calibration | At least five concentrations prepared from certified amino-acid standards |
| Calibration acceptance | R² ≥ 0.995 |
| Identification | Comparison of sample and standard retention times |
| Quantification | External calibration based on analyte peak area |
| Injection repeatability | Peak-area RSD ≤5% |
| Spike-recovery acceptance | 80–120% |

**Gradient-elution program**

| **Time (min)** | **Mobile phase A (%)** | **Mobile phase B (%)** |
| --- | --- | --- |
| 0.0 | 100 | 0 |
| 1.9 | 100 | 0 |
| 18.1 | 43 | 57 |
| 18.6 | 0 | 100 |
| 22.3 | 0 | 100 |
| 23.2 | 100 | 0 |
| 26.0 | 100 | 0 |

B

A

C

**Figure S1:** Representative appearance of the three algal biomasses investigated in this study. (A) *Gigartina chamissoi* (syn. *Chondracanthus chamissoi*), a red alga collected from coastal waters near Ancón, Lima; (B) *Monostroma cuaternaria*, a green alga collected from Lake Titicaca, Puno; and (C) *Cladophora glomerata*, a filamentous green alga collected from the Urubamba River, Cusco, Peru
